# Activation of the l-fucose utilization cluster in *Campylobacter jejuni* induces proteomic changes and enhances Caco-2 cell invasion and fibronectin binding

**DOI:** 10.1016/j.heliyon.2024.e34996

**Published:** 2024-07-26

**Authors:** Pjotr S. Middendorf, Lucas M. Wijnands, Sjef Boeren, Aldert L. Zomer, Wilma F. Jacobs-Reitsma, Heidy M.W. den Besten, Tjakko Abee

**Affiliations:** a Food Microbiology, Wageningen University and Research, Wageningen, the Netherlands; b National Institute for Public Health and the Environment, Bilthoven, the Netherlands; c Laboratory of Biochemistry, Wageningen University and Research, Wageningen, the Netherlands; d Faculty of Veterinary Medicine, Department of Infectious Diseases and Immunology, Utrecht University, Utrecht, the Netherlands; e WHO Collaborating Center for Campylobacter/OIE Reference Laboratory for Campylobacteriosis, Utrecht, the Netherlands

**Keywords:** Pathogen, Survival, Secretion system, Lipooligosaccharides, Food safety

## Abstract

Most *Campylobacter jejuni* isolates carry the fucose utilization cluster (*Cj0480c-Cj0489*) that supports the metabolism of l-fucose and d-arabinose. In this study we quantified l-fucose and d-arabinose metabolism and metabolite production, and the impact on Caco-2 cell interaction and binding to fibronectin, using *C. jejuni* NCTC11168 and the closely related human isolate *C. jejuni* strain 286. When cultured with l-fucose and d-arabinose, both isolates showed increased survival and production of acetate, pyruvate and succinate, and the respective signature metabolites lactate and glycolic acid, in line with an overall upregulation of l-fucose cluster proteins. *In vitro* Caco-2 cell studies and fibronectin-binding experiments showed a trend towards higher invasion and a significantly higher fibronectin binding efficacy of *C. jejuni* NCTC11168 cells grown with l-fucose and d-arabinose, while no significant differences were found with *C. jejuni* 286. Both fibronectin binding proteins, CadF and FlpA, were detected in the two isolates, but were not significantly differentially expressed in l-fucose or d-arabinose grown cells. Comparative proteomics analysis linked the *C. jejuni* NCTC11168 phenotypes uniquely to the more than 135-fold upregulated protein Cj0608, putative TolC-like component MacC, which, together with the detected Cj0606 and Cj0607 proteins, forms the tripartite secretion system MacABC with putative functions in antibiotic resistance, cell envelope stress response and virulence in Gram negative pathogenic bacteria. Further studies are required to elucidate the role of the MacABC system in *C. jejuni* cell surface structure modulation and virulence.

## 1 Introduction

*Campylobacter* is the leading cause of bacterial foodborne gastroenteritis in the world and its prevalence has increased over the past years [[1], [2], [3], [4]]. Clinically, the most relevant species is *Campylobacter jejuni*, responsible for 80–90 % of all diagnosed *Campylobacter* infections [1]. Most infections are linked to consumption of meat products (predominantly poultry), direct contact with animals, and contact with environmental waters [[5], [6], [7], [8], [9], [10]]. *Campylobacter* infection, or campylobacteriosis, generally causes symptoms like watery or bloody diarrhea accompanied by abdominal pain, nausea and fever [11,12]. In a small number of patients, post-infection complications such as the neurological disorder Guillain-Barré syndrome can occur [[13], [14], [15]].

*C. jejuni* is able to colonize the human intestine and has a relatively low dose-response correlation [16,17]. *In vitro*, *C. jejuni* can adhere, invade and survive in human intestinal epithelial cells with a complex array of fitness and virulence factors. The use of genome sequencing techniques has already resulted in identification of several genes encoding putative adhesins, proteases, autotransporters, chemotaxis proteins and cytolethal distending toxin (CDT) [[18], [19], [20], [21]]. Fibronectin is an extracellular glycoprotein on intestinal epithelial cells (IECs) and functions as an adhesion site for *Campylobacter* cells [22]. The highly conserved *C. jejuni* fibronectin-like protein A (FlpA) and fibronectin-binding protein (CadF) have been found to be important for adhesion to human IECs and colonization of chicken [[22], [23], [24]]. However, due to the genome plasticity of the *Campylobacter* species [25], the identification of novel key factors that play a role in *Campylobacter* virulence remains a great challenge [21,26].

*C. jejuni* may enhance its competitive fitness following rewiring its metabolic repertoire during colonization and infection. Next to utilizing citric acid intermediates, amino acids and peptides, some *C. jejuni* isolates have been described to utilize l-fucose, d-arabinose and/or d-glucose as carbon and energy sources [[27], [28], [29], [30], [31], [32], [33], [34], [35]]. Comparative whole genome sequencing (WGS) analysis revealed that approximately 65 % of the sequenced *C. jejuni* isolates possess the so-called l-fucose utilization cluster, designated *Cj0480c* – *Cj0489*, from here on called fuc + isolates. Upon consumption of l-fucose, fuc + isolates have shown increased growth, increased survival, decreased biofilm formation and changes in the general metabolism [28,31,33,36]. Recently it was discovered that except for l-fucose, also d-arabinose is metabolized by the l-fucose utilization cluster and is able to promote growth [28]. The l-fucose utilization cluster is regulated by *Cj0480c* and contains two predicted transporters encoded by *Cj0484* and *Cj0486/FucP*. Following transport of l-fucose through the cell membrane, l-fucose is further metabolized to the end products pyruvic acid and lactic acid via metabolic enzymes *Cj0488*, *Cj0485* (FucX), *Cj0487*, *Cj0482/Cj0483*, *Cj0481* (DapA) and *Cj0489*, respectively [28]. Both pyruvate and lactate can be further metabolized and support growth of *C. jejuni* [33,37]. l-fucose is abundant in host gut mucosal surfaces and incorporated in a variety of fucosylated structures. In Caco-2 cells, type 2 α1,2-fucosylated glycans are evenly distributed over the cell surface, and type 1 α1,2-fucosylated glycans structures were found as membrane-bound mucin patches [38]. Since *C. jejuni* lacks fucosidases, it is not able to release l-fucose from glycosylated host proteins [28,39]. However, recent co-culture studies with the gastrointestinal bacterium *Bacteroides fragilis* provided evidence that an hyperinvasive *C. jejuni* isolate was able to utilize the l-fucose released by *B. fragilis* fucosidases, and show enhanced invasiveness towards human Caco-2 cells [38].

Despite the influence of l-fucose on growth, survival and its effect on the metabolism of *C. jejuni*, relatively little is known about the role of the l-fucose utilization clusters and the impact on virulence in fuc + *C. jejuni* isolates. Here we report a comparative analysis of l-fucose and d-arabinose utilization capacity and *in vitro* Caco-2 cell adhesion/invasion and fibronectin binding efficacy of the model strain *C. jejuni* NCTC11168 and the closely related human isolate *C. jejuni* 286. Correlating observed metabolic and *in vitro* virulence phenotypes of the two *C. jejuni* strains to proteomic profiles will provide insight in strain-specific differences in activation of l-fucose and d-arabinose metabolic enzymes and impact on physiology and *in vitro* virulence.

## 2 Methods

### 2.1 Bacterial isolate selection and medium preparation

A phylogenetic tree based on alignments of 128 human *C. jejuni* isolates, obtained via the Dutch National Institute for Public Health and the Environment (RIVM) and described in Ref. [40], was made using Roary [41], MAFFT (https://mafft.cbrc.jp/alignment/software/) and fasttree software http://www.microbesonline.org/fasttree/). The presented dataset in this study can be found in online repositories of BioProject: https://www.ncbi.nlm.nih.gov/, PRJEB38253, the accession number of isolate *C. jejuni* 286 within this BioProject is: ERR4224289. Visualization was done by using ITOL (itol.embl.de). Based on this phylogenetic tree, the reference isolate *C. jejuni* NCTC11168 (isolated from human faeces) described in our previous study [36] and the closely related isolate *C. jejuni* 286, isolated from an hospitalized patient, were selected (Suppl. Fig. 1).

*Campylobacter* cultures were obtained on Columbia Agar Base (CAB, Oxoid, Landsmeer, the Netherlands) plates supplemented with 5 % (v/v) lysed horse blood (BioTrading Benelux B.V. Mijdrecht, the Netherlands) and 0.5 % (w/w) bacteriological agar No.1 (Oxoid). *Campylobacter* stock cultures were prepared by growing a selected colony in 10 mL BactoTM Heart Infusion broth (Becton, Dickinson and Company, Vianen, the Netherlands) for 24 h at 41.5 °C in microaerobic conditions (5 % O_2_, 10 % CO_2_, 85 % N_2_), which was created using an Anoxomat WS9000 (Mart Microbiology, Drachten, the Netherlands). Glycerol stocks were prepared using 30 % (v/v) glycerol (Fluka) and 70 % overnight culture and were stored at −80 °C.

Working cultures were prepared by streaking a loopful of the *Campylobacter* freezer stocks on CAB plates, and plates were incubated for 48 h at 41.5 °C in microaerobic conditions. A single colony was routinely selected and inoculated in 10 mL MEMα (Minimal Essential Medium alpha) medium (Thermo Fisher Scientific, Bleiswijk, the Netherlands) supplemented with 20 μM FeSO_4_ (Merck, Schiphol-Rijk, the Netherlands), and the culture was cultured overnight at 41.5 °C microaerobically. A second overnight culture was made by diluting the *Campylobacter* culture 1:100 (v/v) in 10 mL fresh MEMα medium. The culture was incubated at 41.5 °C for 24 h in microaerobic conditions to obtain standardized working cultures.

Prior to the experiments, MEMα medium was prepared without and with supplementation of 25.0 mM l-fucose (MEMαF medium) or 25.0 mM d-arabinose (MEMαA medium) and filter-sterilized using 0.2-μm pore sized filters (Fisher Scientific). Sterilized infusion bottles, closed using a rubber stopper and aluminium cap, were filled with 45 mL filter-sterilized MEMα medium, MEMαF medium or MEMαA medium by using a syringe. Filled infusion bottles were stored at 4 °C until further use.

### 2.2 l-fucose and d-arabinose growth experiments

As described previously [36], working cultures were decimally diluted in MEMα medium to a cell concentration of approximately 5.0 log_10_ CFU/mL. A final dilution step was done by adding 5 mL into the infusion bottles filled with 45 mL MEMα medium, MEMαF medium or MEMαA medium, resulting in a starting cell concentration of 4.0 log_10_ CFU/mL. Incubation of the inoculated infusion bottles was done at 37 °C microaerobically. At various timepoints, namely, day 0, day 1, day 2, day 3 day, 4 and day 7, approximately 4 mL sample was taken from each infusion bottle. After each sampling, the head space of infusion bottles was flushed for 2 min with microaerobic gas (5 % O_2_, 10 % CO_2_, 85 % N_2_) using a home-made gas flushing device using syringes to puncture the rubber stopper.

The 4 mL-aliquots were used to determine the bacterial concentration. The remainder of each sample (in either MEMα medium, MEMαF medium or MEMαA medium) was stored at −20 °C for high performance liquid chromatography (HPLC) analyses. Bacterial concentrations were determined by decimally diluting 1 mL of sample in peptone physiological salt solution (PPS, Tritium Microbiologie, Eindhoven, the Netherlands), followed by plating on CAB plates. CAB plates were incubated in air-tight jars for 48 h at 41.5 °C in microaerobic conditions. Colonies were counted and expressed in log_10_ CFU/mL. Each sample was microscopically analysed using an Olympus BX 41 microscope (lens Ach 100x/1.25, Olympus Nederland, Leiderdorp, the Netherlands) and pictures were captured using CellSens Imaging software (Olympus Corporation). Three biologically independent reproductions were performed per condition, i.e., cells cultured in MEMα medium, MEMαF medium and MEMαA medium, on different days.

### 2.3 High performance liquid chromatography for organic acids and amino acids

The frozen samples stored during the growth experiments were thawed for HPLC analyses using the procedure as previously described by Middendorf et al. (2022) [36]. Briefly, For organic acids analysis, collected samples were centrifugated at 13,000 *g* at 4 °C for 5 min. Pellets were removed and the supernatant was treated for protein decontamination with Carrez A (K_4_FeCN)6·3H_2_0, Merck) and B (ZnO_4_·7H_2_0, Merck). After centrifugation, the supernatant was added to HPLC vials. Quantitative analyses were done using standards with pre-made concentrations for l-fucose, d-arabinose, acetate, alpha-ketoglutarate, succinate, glycolic acid, pyruvate and lactate. The HPLC was performed on an Ultimate 3000 HPLC (Dionex, Sunnyvile, USA) equipped with an RI-101 refractive index detector (Shodex, Kawasaki, Japan), an autosampler and an ion-exclusion Aminex HPX – 87H column (7.8 × 300 mm) with a guard column (Bio-Rad, Hercules, CA, USA). As mobile phase, 5 mM H_2_SO_4_ (Merck) was used at a flow rate of 0.6 mL/min. Column temperature was kept at 40 °C. For each run, the injection volume was 10 μL and the run time 30 min. Chromeleon software (Thermo Fisher Scientific, Waltham, USA) was used for quantification of compound concentrations. HPLC analyses confirmed the absence of l-fucose and d-arabinose in MEMα medium.

For amino acids analyses, samples were used in aliquots of 40 μL. These aliquots were kept on ice and were diluted with 50 μL of 0.1 M HCl (containing 250 μM Norvalin as internal standard, Merck). The samples were deproteinized by addition of 10 μL of cold 5-sulphosalicilic acid (SSA, Merck) (300 mg/mL) and centrifuged at 13,000 *g* at 4 °C for 10 min. In order to obtain an optimal pH for derivatization (pH between 8.2 and 10.0), approximately 60–150 μL of 4N NaOH was added to 5 mL of the AccQ•Tag™ Ultra borate buffer (Borate/NaOH buffer, Waters, Milford, USA). For derivatization 60 μL of Borate/NaOH was added to a total recovery vial. Twenty μL of the supernatant obtained after deproteinization of the plasma was added and mixed. To each of the vials 20 μL of AccQ Tag Ultra derivatization reagent (Waters) dissolved in acetonitrile was added and mixed for 10 s. Each vial was immediately capped. The vials were then heated for 10 min at 55 °C. Quantitative analyses were done using standards with pre-made concentrations for, histidine, asparagine, serine, glutamine, arginine, glycine, aspartic acid, glutamic acid, threonine, alanine, proline, cysteine, lysine, tyrosine, methionine, and valine. HPLC was performed on an Ultimate 3000 HPLC (Dionex) equipped with an RI-101 refractive index detector (Shodex), an autosampler and an ion-exclusion Aminex HPX – 87H column (7.8 × 300 mm) with a guard column (Bio-Rad). As mobile phase, eluents A and B (Waters) was used at a flow rate of 0.7 mL/min. Column temperature was kept at 55 °C. For each run, the injection volume was 1 μL and the run time 17 min. Chromeleon software (Thermo Fisher Scientific) was used for the determination of compound concentrations. Baseline separation was obtained for all amino acids used except glutamine and arginine.

### 2.4 *C. jejuni* adhesion and invasion of Caco-2 cells

To establish a confluent monolayer of Caco-2 cells, production of differentiated Caco-2 cells (human intestinal epithelial cells, ATCC HTB-37) were carried out by growing Caco-2 cells in 12-well plates (Corning Inc. ID 3513) in Dulbecco's Modified Eagle Medium (no glucose) (DMEM) medium (Gibco, Invitrogen, USA). The 12-well plates were incubated at 37 °C for 12–14 days with the DMEM medium being refreshed every 2 days. For more detail see [42].

Prior to adhesion and invasion of the Caco-2 cells, the standardized working culture of *C. jejuni* was inoculated in MEMα, MEMαF or MEMαA medium aiming for an initial concentration of 3–4 log_10_ CFU/mL, and cells were grown microaerobically for 48 h to reach CFU counts of approximately 8.0 log_10_ CFU/mL to induce l-fucose or d-arabinose utilization, respectively. Next, 40-mL aliquots cultures were spun down for 5 min at 10,000 *g* and were three times washed in Phosphate-buffered saline (PBS). Then the *C. jejuni* cultures were concentrated to a volume of 4 mL reaching 9.0 log_10_ CFU/mL.

For adhesion and invasion experiments, differentiated Caco-2 cells were inoculated to reach a total cell count of 1.6 × 10^5^ cells/well into the 12-well tissue culture plates containing either DMEM, DMEM supplemented with 25 mM l-fucose or DMEM supplemented with 25 mM d-arabinose. Fifty μl of the *C. jejuni* cultures grown in MEMα or in MEMαF or MEMαA medium were inoculated in the 12-well tissue culture plates containing Caco-2 cells, reaching approximately 8.0 log_10_ CFU per well. The 12 well plates were then centrifuged for 1 min at 175 *g* to create a proximity between the Caco-2 and *C. jejuni* cells, after which the 12-wells plates were incubated microaerobically at 37 °C for 3.5 h.

After incubation, the wells were washed three times with PBS using a pipette to remove non-adhered *C. jejuni* cells. Half of the plate was lysed with 1 mL of 1 % (v/v) Triton X-100 in PBS and serially diluted in PBS for quantification of the number of adhered and invaded *C. jejuni* cells. The other half of the plate was subsequently incubated microaerobically for 1 h with 0.3 % gentamicin (Gibco, Invitrogen, USA) to eliminate all extracellular *C. jejuni* cells. Then each well was washed with PBS buffer three times to remove all gentamicin, after which the Caco-2 cells were lysed with 1 mL of 1 % (v/v) Triton X-100 in PBS and serially diluted in PBS for quantification of the number of invaded *C. jejuni* cells. Three biologically independent reproductions were performed per condition.

### 2.5 Fibronectin binding assay

Fibronectin (Fn) binding assays were performed using a previously described method [23]. Flat-bottom 96-well plates (Costar, Corning, NY, USA) were coated with a 1-mg/mL solution of Fn in 0.05 M Tris-buffered saline at pH 7.5 (Sigma, Munich, Germany) and incubated overnight at 4 °C. The *C. jejuni* cultures were grown for 48 h in MEMα, MEMαF or MEMαA medium and the cultures were washed three times using PBS and were resuspended in PBS to reach a final concentration of approximately 8.0 log_10_ CFU/mL. The Fn-coated 96 wells plate was rinsed three times with PBS, and 100 μl of bacterial suspensions were added to each well and incubated in microaerobic conditions at 37 °C for 1 h After incubation, the wells were washed three times with 100 μl PBS per well, and adhered bacteria were removed by the addition of 100 μL Tryple™ Express (Gibco, Invitrogen). To enumerate the number of adhered bacteria, serial dilutions of the Tryple suspension were made in MEMα medium, plated on CAB plates and colonies were counted after 48 h incubation at 41.5 °C in microaerobic conditions. Three biologically independent reproductions were performed per condition.

### 2.6 Statistical analyses

Biological reproductions, each consisting out of two or three technical replicates, were used to statistically test the differences in log_10_-counts observed between two conditions, such as growth in MEMα medium and growth in MEMαF medium. These differences were statistically tested using a two-tailed Student's t-test. P values ≤ 0.05 were considered as significant difference.

### 2.7 Proteomics

The working cultures of *C. jejuni* were used to inoculate in triplicate infusion bottles to a concentration of 5.0 log_10_ CFU/mL that were filled with 45 mL of MEMα, MEMαF or MEMαA medium, and cultures were grown for 48 h at 37 °C in microaerobic conditions. Thirty mL of each culture was centrifugated and washed three times in TRIS buffer (pH 8.0) and resuspended in a final volume of 100 μl. The cultures were then sonicated with a sonication probe for 30 s. Protein concentrations were determined by the bicinchoninic acid (BCA) assay.

The protein aggregation capture (PAC) method as described by Refs. [43,44] was used in a slightly modified way for sample preparation for proteomics analysis. Briefly, each sample, containing 60 μg of protein, was reduced with 15 mM DTT at 45 °C for 30 min, unfolded in 6 M urea and alkylated with 20 mM acrylamide at room temperature for 30 min. The pH of the protein solution was adjusted to 7 using 10 % (v/v) trifluoro-acetic acid (TFA). SpeedBeads (magnetic carboxylate modified particles, GE Healthcare, Chicago, USA) of products 45152105050250 and 65152105050250 were mixed with 1:1 ratio at 50 μg/μl, and 8 μl of SpeedBeads was added to each protein sample. Acetonitrile was added up to 71 % (v/v) to the protein beads mixture, incubated at room temperature with gentle shaking for 20 min. A magnet was used to separate the SpeedBeads from the supernatant for 30 s, and the supernatant was removed. The SpeedBeads were then washed with 1 mL of 70 % ethanol and 1 mL of 100 % acetonitrile successively, resuspended in 100 μl of 5 ng/μl sequencing grade trypsin solution in 50 mM ammonium bicarbonate and incubated overnight at room temperature with gentle shaking. The pH of SpeedBeads suspension was adjusted to 3 using 10 % TFA, and the SpeedBeads were separated from the supernatant by using a magnet. The supernatant was filtered using C8 Empore disk filters. To improve yield, 0.1 % formic acid was used to wash the beads and a 1:1 (v/v) mixture of acetonitrile and 0.1 % formic acid was used to wash the filter. All eluents were combined and dried to 10–15 μl, then topped up to 50 μl with 0.1 % formic acid.

For the LC-MS/MS analysis, 5 μl of prepared sample was injected into the system, and the analysis was performed as described in Ref. [44]. The MaxQuant quantitative proteomics software package was used to analyse LC-MS data with all MS/MS spectra as described by Ref. [45] and the proteome of *C. jejuni* NCTC11168 (UniProt ID UP000000799) was used as the protein database. Next, Perseus was used for filtering and further bioinformatics and statistical analysis of the MaxQuant ProteinGroups files [46]. Reverse hits were removed; identified protein groups contained minimally two peptides, of which at least one is unique and one unmodified. The normalised label-free quantification (LFQ) intensity values as calculated by MaxQuant of each tested condition measured in biological triplicates were used.

The lower detection limit was set at 5.6 log_10_ (LFQ protein abundance), just below the lowest measured LFQ intensity by imputation NaN's by 5.6 after logarithmization. T-tests were performed on triplicates of each tested condition and were corrected with a false-discovery rate of 0.05.

Protein fold-changes were calculated as actual fold change, as example: a fold-change of 2 indicates that a two times higher protein concentration was measured in the tested condition versus the reference condition; and a fold-change of −2 indicates that a two times lower protein concentration was observed in the tested condition versus the reference condition. The minus fold-change was calculated by translating the initial -log_10_ value to a positive number and calculating the fold-change of this positive log_10_ value, which was finally converted to a minus fold-change.

Proteins were only considered to be differentially expressed when a significant fold-change of over 1.5 or below −1.5 was calculated.

## 3 Results

### 3.1 Effect of l-fucose and d-arabinose on the growth of *C. jejuni* NCTC11168 and *C. jejuni* 286 and survival in MEMα medium

**Fig. 1:**
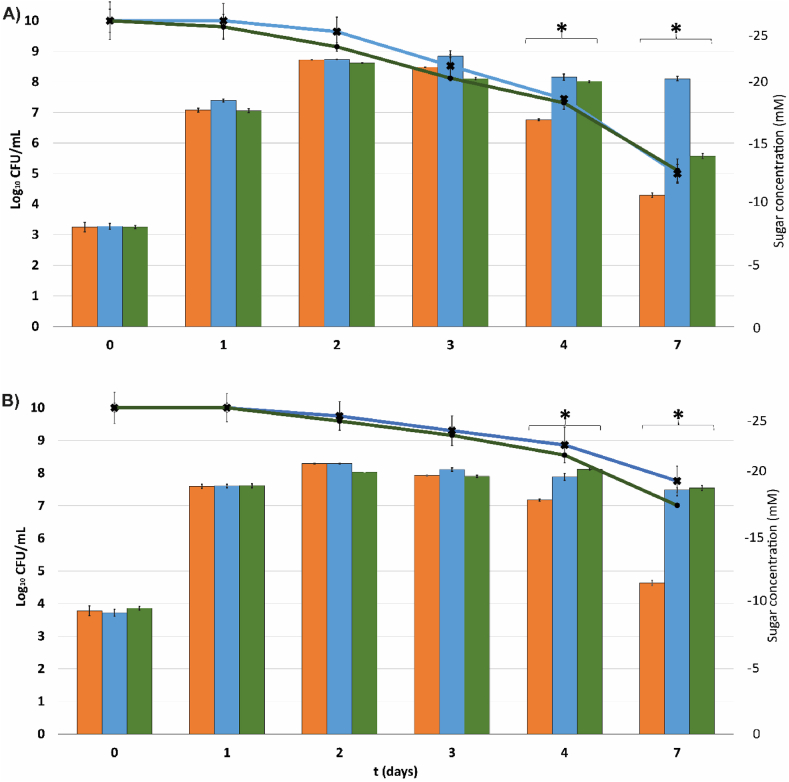
Growth of two human *C. jejuni* isolates in MEMα medium (orange bars), MEMαF medium (blue bars) and MEMαA medium (green bars). A) Growth of *C. jejuni* NCTC11168. B) Growth of *C. jejuni 286*. The blue and green lines indicate l-fucose and d-arabinose concentrations in the medium, respectively. Significant differences in log_10_ CFU counts/mL between MEMαF medium and MEMα medium, and between MEMαA medium and MEMα medium are indicated with an asterisk.

Growth and survival of *C. jejuni* NCTC11168 and *C. jejuni* 286 was investigated in MEMα, MEMαF and MEMαA medium up to 7 days (Fig. 1). At day 1, 2 and 3, no significant differences in growth were observed in the tested media, with CFU counts reaching up to 8.7 ( ± 0.1) and 8.2 ( ± 0.1) log_10_ CFU/mL for *C. jejuni* NCTC11168 and *C. jejuni* 286, respectively. From day 4 onwards, cell counts in MEMα medium decreased, reaching 4.2 and 4.6 log_10_ CFU/mL at day 7 for isolates *C. jejuni* NCTC11168 and *C. jejuni* 286, respectively. In MEMαF medium, CFU counts were more stable overtime, slightly decreasing to final cell concentrations of 8.1 and 7.5 log_10_ CFU/mL for strain *C. jejuni* NCTC11168 and *C. jejuni* 286, respectively. In MEMαA medium, CFU counts decreased after day 4 for isolate *C. jejuni* NCTC11168, while *C. jejuni* 286 CFU counts remained more stable, reaching 5.5 and 7.5 log_10_ CFU/mL at day 7, respectively.

Incubation of the two *C. jejuni* strains in MEMαF and MEMαA medium showed that l-fucose and d-arabinose concentrations started to decrease at day 2, pointing to activation of metabolism. For *C. jejuni* NCTC11168 similar l-fucose and d-arabinose consumption patterns were observed and final consumed concentrations were approximately 12.5 mM at day 7, while CFU counts at day 7 were highest in MEMαF medium. For isolate *C. jejuni* 286, slightly lower consumption of l-fucose and d-arabinose compared to *C. jejuni* NCTC11168 was observed, with an approximate 6.0 mM and 7.5 mM consumption at day 7, respectively.

### 3.2 Effect of l-fucose and d-arabinose on the metabolism of *C. jejuni* NCTC11168 and *C. jejuni* 286

**Fig. 2:**
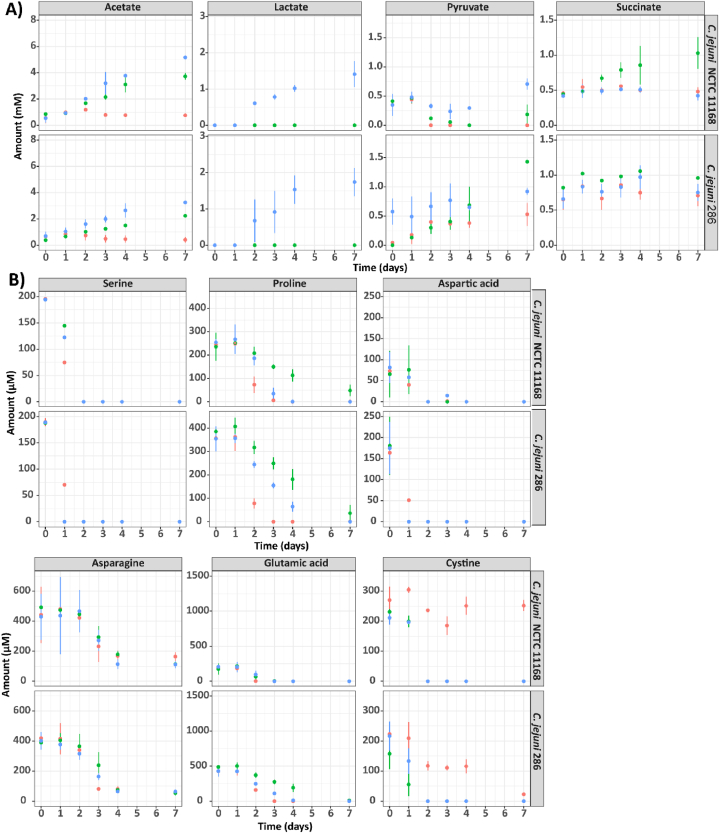
HPLC quantifications of *C. jejuni* NCTC11168 and *C. jejuni* 286 in MEMα medium (red dots), MEMαF medium (blue dots) or MEMαA medium (green dots). A) Organic acids. B) Amino acids. Each value represents the average of three biologically independent replicates, and error bars show the standard deviation.

We determined the concentrations of selected compounds which were previously found to be utilized or produced during *C. jejuni's* l-fucose metabolism [36] (Fig. 2). A schematic overview of uptake and metabolism uniquely coupled to l-fucose or d-arabinose is presented in Suppl. Fig. 2. Activation of the l-fucose pathway via l-fucose or d-arabinose metabolism was confirmed by measuring the respective signature products, lactic acid and glycolic acid (Fig. 2, Suppl. Fig. 3).

Acetate production was observed for *C. jejuni* NCTC11168 and *C. jejuni* 286 in all tested media and was highest in the presence of l-fucose (5.22 and 3.25 mM), followed by d-arabinose (3.58 and 2.23 mM), in comparison with MEMα medium (0.72 and 0.41 mM) (Fig. 2). For lactic acid, final concentrations were 1.59 mM and 1.74 mM in MEMαF medium at day 7 for isolate *C. jejuni* NCTC11168 and *C. jejuni* 286, respectively. Pyruvate was first consumed by *C. jejuni* NCTC11168, while concentrations increased at a later stage reaching 0.70 mM and 0.26 mM at day 7 in MEMαF and MEMαA medium, respectively. For isolate *C. jejuni* 286, pyruvate was produced in all tested media, reaching a final concentration of 0.54 mM, 0.92 mM and 1.41 mM in MEMα, MEMαF and MEMαA medium, respectively. No changes in succinate concentrations were observed for *C. jejuni* NCTC11168 and *C. jejuni* 286, when grown in MEMα and MEMαF medium, while succinate concentration increased when grown in MEMαA medium, reaching final concentrations of 1.19 mM and 0.96 mM for *C. jejuni* NCTC11168 and *C. jejuni* 286, respectively.

As expected, a strong preference was observed for the substrate serine, which was fully depleted in all tested media in two days by isolate NCTC11168 and in one day by isolate *C. jejuni* 286 (Fig. 2B). Both isolates also fully depleted proline by day 3 in MEMα medium and by day 7 in MEMαF medium. No full depletion was observed in MEMαA medium, where proline was consumed until day 7, reaching a final concentration of 48.15 μM and 48.60 μM for isolate *C. jejuni* NCTC11168 and *C. jejuni* 286, respectively. Aspartic and glutamic acid were fully depleted on day 2 in all tested conditions, whereas asparagine concentrations decreased the first 4 days and remained constant at low levels of approximately 0.15 mM in all tested media. In MEMαF and MEMαA media cystine was fully depleted on day 2 in both tested isolates, while no depletion (isolate *C. jejuni* NCTC11168) or delayed depletion (isolate *C. jejuni* 286) was observed in MEMα medium.

### 3.3 Adhesion and invasion of Caco-2 cells and binding to fibronectin with l-fucose and d-arabinose grown cells

**Fig. 3:**
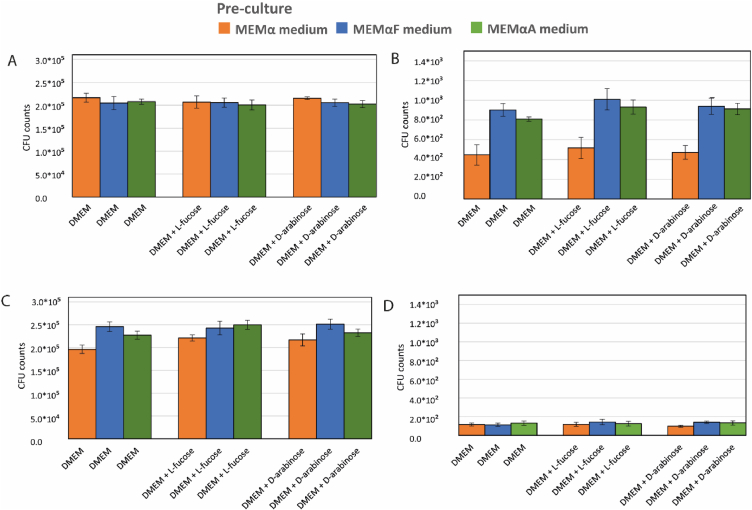
Caco-2 cell adhesion and invasion results of *C. jejuni* NCTC11168 and *C. jejuni* 286 cells that were precultured for two days in MEMα medium (orange bars), MEMαF medium (blue bars) or MEMαA medium (green bars) and incubated in DMEM, DMEM + l-fucose or DMEM + d-arabinose. Adhesion (A) and invasion (B) of *C. jejuni* NCTC11168, and adhesion (C) and invasion (D) of *C. jejuni* 286. Error bars indicate the standard deviation of three technical replicates.

The impact of pre-activation of the l-fucose utilization cluster on adhesion and invasion of Caco-2 cells was assessed in DMEM medium, DMEM-F medium and DMEM-A medium, as this medium is commonly used during invasion assays.

l-fucose and d-arabinose preculturing did not affect the adhesion efficacy for both isolates, neither the supplementation of the adhesion medium with l-fucose or d-arabinose (Fig. 3A and C, Suppl. Fig. 4). On the other hand, an increasing trend was observed for the invasion capacity of *C. jejuni* NCTC11168 upon pre-activation of the l-fucose cluster by l-fucose or d-arabinose preculturing, while supplementation of the invasion medium with l-fucose or d-arabinose did not affect invasion efficacy (Fig. 3B, Suppl. Fig. 4). Notably, *C. jejuni* 286 showed no significant differences in invasion capacity when precultured with l-fucose or d-arabinose (Fig. 3D, Suppl. Fig. 4).

**Fig. 4:**
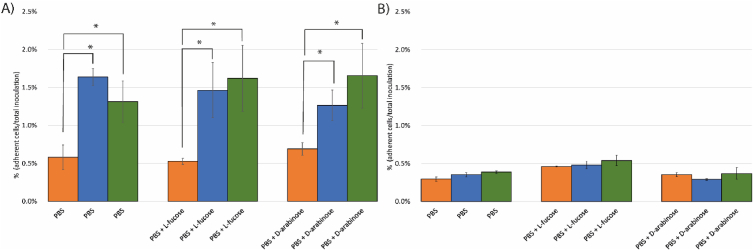
Fibronectin binding of *C. jejuni* NCTC11168 and *C. jejuni* 286 that were precultured for two days in MEMα medium (orange bars), MEMαF medium (blue bars) or MEMαA medium (green bars). and incubated in PBS, PBS supplemented with l-fucose, and PBS supplemented with d-arabinose. A) fibronectin binding of *C. jejuni* NCTC11168. B) and of *C. jejuni* 286. Data from three biological replicates; asterisks indicate significant difference with the cells cultured in non-supplemented medium (P < 0.005).

Next, the effect of l-fucose and d-arabinose pre-culturing on binding to fibronectin was investigated. Following an 1 h incubation of isolate *C. jejuni* NCTC11168, a significantly higher number of cells were bound when cells were precultured with l-fucose or d-arabinose compared to the control cells (Fig. 4A). *C. jejuni* 286 showed low fibronectin binding capacity and no significant differences were observed in the tested conditions (Fig. 4B). In all cases similar results were obtained after extending the incubation time up to 3.5 h (data not shown).

### 3.4 Significant up and down regulation of proteins after activation of the l-fucose utilization cluster

In total, 88 proteins were significantly differentially expressed in the two strains when cultured in MEMαF and MEMαA compared to MEMα medium (Supp. Table 1). Nine of these proteins were upregulated in all tested conditions; eight of them belonged to the l-fucose utilization cluster (Cj0481, Cj0482, Cj0483, Cj0485, Cj0486, Cj0487, Cj0488, Cj0489), and one was a membrane related protein (Cj0771c). Furthermore, two proteins were downregulated in all tested conditions, namely, the bifunctional protein PutA, and putative periplasmic protein Cj0200c, a member of the two-component system DccRS (Cj1223c-Cj1222c) regulon [47]. PutA is a proline dehydrogenase which oxidizes L-proline to L-glutamate, and is conceivably linked to the observed reduced proline consumption in MEMαF and MEMαA medium compared to MEMα medium.

### 3.5 Putative virulence associated proteins

The analysis of presence and significantly differentially expressed putative virulence factors in *C. jejuni* NCTC11168 and 286 was based on the list of 236 putative *C. jejuni* virulence proteins presented in Suppl. Table 2, which was extracted from the *virulence factors of pathogenic bacteria* database (http://www.mgc.ac.cn/VFs/) and extended with information from selected publications on *Campylobacter* virulence [[48], [49], [50], [51], [52], [53]]. Notably, in total 177 and 166 of these 236 putative virulence factors were detected in our proteomics analysis (Suppl. Table 2), and of these, eight and nine were significantly up or down regulated in the presence of l-fucose and/or d-arabinose in *C. jejuni* NCTC11168 and *C. jejuni* 286, respectively (Table 1).

**Table 1:** Presence and up regulation of l-fucose utilization cluster proteins. Values indicate a fold-change in comparison with MEMα medium. ND, not detected.

Protein	Function	NCTC11168 fuc vs ref	NCTC11168 ara vs ref	286 fuc vs ref	286 ara vs ref
Cj0481|Q0PB32	Dihydrodipicolinate synthase	23.6	12.4	19.5	11.8
Cj0482|Q0PB31	Altronate hydrolase/dehydratase	273	160	152	101
Cj0483|Q0PB30	Altronate hydrolase/dehydratase	512	253	36.2	34.2
Cj0484|Q0PB29	MFS transporter	ND	ND	102	69.2
Cj0485|Q0PB28	Short chain dehydrogenase	23.6	23.6	17.4	13.3
Cj0486|Q0PB27	MFS transporter	46.4	21.8	19.9	15.2
Cj0487|Q0PB26	Amidohydrolase	88.0	39.6	9.0	8.6
Cj0488|Q0PB25	Epimerase	70.3	39.1	140	93.3
Cj0489|Q0PB23	Aldehyde dehydrogenase	3.0	2.0	26.6	18.1

From these proteins, only one putative virulence protein, Cj0608, was uniquely upregulated in MEMαF and MEMαA grown cells of *C. jejuni* NCTC11168, and therefore conceivably linked to the observed enhanced Caco-2 invasion and fibronectin binding phenotypes of these cells. Cj0608 is a putative outer membrane protein, part of the MacABC complex (*Cj0606-Cj0608*), a putative Type 1 secretion system, linked to the DccSR regulon.

Additionally, several significant differences in protein expression were observed that could not directly be linked to our observed invasion and fibronectin binding phenotype. Of these virulence proteins, three were downregulated in isolate *C. jejuni* 286, but not in isolate *C. jejuni* NCTC11168, namely, Peb3 (adhesin), FlgD (basal-body rod modification protein) and Cj1169c (putative periplasmic protein) [22,54,55]. Furthermore, the upregulation of Cj0371 in isolate *C. jejuni* 286, but not in isolate *C. jejuni* NCTC11168, potentially influences the virulence of this isolate, as activation of this protein has a negative role on *Campylobacters* chemotaxis [56].

Notably, CadF and FlpA, involved in binding to fibronectin, were detected in both isolates, but not significantly differentially expressed in the presence of l-fucose or d-arabinose. Interestingly, the protein WlaN/Cj1139c, Beta-1,3 galactosyltransferase, which is only produced in *C. jejuni* NCTC11168, is co-expressed with the fibronectin binding protein CadF (string-db.org), suggesting a possible link towards the increased fibronectin binding in this isolate. Four other virulence proteins were present in *C. jejun*i NCTC11168, that were absent in *C. jejuni* 286, namely**,** FlgB, PseD, PglC and Cj1337c. Furthermore, one protein was absent in *C. jejuni* NCTC11168 and present in *C. jejuni* 286, namely, CipA.

Moreover, in the presence of either l-fucose or d-arabinose, several proteins were uniquely significantly differentially expressed in isolate *C. jejuni* NCTC11168. In the presence of l-fucose, two proteins were uniquely upregulated, catalase KatA, involved in H_2_O_2_ detoxification (survival), and capsular glycosyltransferase PglC, involved in cell wall synthesis, while another capsular protein, Cj1417c (labelled as immune modulation) was downregulated. In the presence of d-arabinose, CdtB, an exotoxin protein, and Cj0125c, encoding a DksA-like protein, were uniquely upregulated, while Cj0358, a putative cytochrome peroxidase protein, was downregulated. Additionally, a number of putative virulence proteins were uniquely upregulated in the presence of l-fucose in *C. jejuni* 286, including exotoxin CdtC and the enterochelin uptake periplasmic binding protein CeuE. In the presence of d-arabinose, CmeA, a subunit of multidrug efflux system CmeABC, was upregulated, while Peb1A, a major host cell-binding factor protein, was downregulated.

## 4 Discussion

The l-fucose utilization cluster is present in the majority of *Campylobacter jejuni* and *Campylobacter coli* isolates and its involvement in a range of parameters including growth and virulence has been reported [[31], [32], [33],^38^]. Via the l-fucose utilization cluster, both l-fucose and d-arabinose can be metabolized, however, l-fucose is the preferred carbon source over d-arabinose [28]. In the current study we provided novel insights in the l-fucose utilization cluster on protein level and linked this to phenotypic observations. Our proteomic analyses of two selected fuc + isolates; reference isolate *C. jejuni* NCTC11168 (human stool isolate) and isolate *C. jejuni* 286 (hospitalized patient isolate), showed significant upregulation of proteins in the l-fucose utilization cluster when cells were grown in the presence of l-fucose or d-arabinose compared to the reference condition. Furthermore, the previously described putative transporter Cj0484 [31,33], was not detected in *C. jejuni* NCTC11168, indicating that it is not functional in this strain in the tested conditions. Interestingly, Cj0484 was detected and highly upregulated in *C. jejuni* 286, suggesting a function in this isolate that remains to be elucidated.

In the current study, comparative growth analysis of the two isolates showed comparable growth in all tested media up to day 3, with *C. jejuni* NCTC11168 and *C. jejuni* 286 reaching maximum CFU/mL of 8.7 log_10_ and 8.2 log_10_, respectively. The l-fucose metabolism and growth data of isolate *C. jejuni* NCTC11168 are in line with previous growth performance and increased survival observed for this isolate [36]. HPLC analyses showed a higher l-fucose consumption for isolate *C. jejuni* NCTC11168, in comparison with isolate *C. jejuni* 286, which could be linked to the higher fold expression-of several proteins in the l-fucose utilization cluster, including the transporter Cj0486, the amidohydrolase Cj0487 and the altronate hydrolases Cj0482 and Cj0483. Further HPLC analyses confirmed that the amino acids serine, aspartic and glutamic acid were depleted at day 3 in MEMα, MEMαF and MEMαA medium, in line with previous studies that suggested that these amino acids are preferred substrates for growth of *Campylobacter* [28,36]. In addition, both tested isolates depleted proline more rapidly in the absence of l-fucose or d-arabinose, in line with the observed downregulation of PutA (Suppl. Table 1), previously described to play an essential role in proline metabolism [57].

Next, we investigated whether activation of the fucose utilization clusters in the selected *C. jejuni* NCTC11168 and 286 strains affected their interaction with Caco-2 cells and their binding to fibronectin. Using preactivated cells harvested at day 2 from cultures grown in MEMα without and with l-fucose or d-arabinose, an approximate two-fold higher invasion efficacy of Caco-2 cells was observed for *C. jejuni* NCTC11168 when grown with added substrates. Additionally, these preactivated *C. jejuni* 11,168 cells showed significantly higher fibronectin binding efficacy compared to control cells. For *C. jejuni* 286 neither enhanced Caco-2 cells invasion nor enhanced binding to fibronectin was observed for pre-activated cells. Our data obtained with pre-activated *C. jejuni* NCTC11168 provide further support for the results presented by Luijkx et al. (2020), who suggested that *C. jejuni* fuc + isolates scavenge and metabolize L‐fucose and alter their invasive properties [38]. Using non-preactivated cells of hyper virulent *C. jejuni* 108 (intestinal isolate from patient with recurrent infections), the authors showed that exogenous l-fucose supplied by action of fucosidases from *B. fragilis*, promoted growth and invasion of Caco-2 cells. Invasion counts of isolate *C. jejuni* 108 were 6000 CFU in DMEM medium and 8000 CFU when l-fucose was added [38]. Our reference isolate, *C. jejuni* NCTC11168, a model strain that was initially isolated from human stool in 1977, showed Caco-2 invasion counts ranging from 400 to 1000 CFU, and as expected, significantly lower than the hyper virulent isolate *C. jejuni* 108 and in line with previous reported efficacies [38,58]. Interestingly, in our study with non-preactivated *C. jejuni* NCTC11168 cells, addition of l-fucose or d-arabinose to the Caco-2 assay did not enhance invasion capacity of the cells. These results suggest that the incubation time of 3.5 h was not sufficient to enable activation of the l-fucose utilization cluster in this strain, which is in line with our current and previously published data [36], that showed onset of l-fucose and d-arabinose metabolism at day 2, after depletion of preferred amino acids including L-serine from the medium. Possible mechanisms underlying differences between *C. jejuni* isolates in transcriptional activation of l-fucose utilization and/or modulation by amino acid metabolism remain to be elucidated.

The current study presented evidence that preactivated cells of *C. jejuni* NCTC11168 grown in MEMαF and MEMαA medium, showed an increasing trend for Caco-2 invasion. Furthermore, for the same isolate a significantly increased fibronectin binding capacity was observed compared to MEMα grown cells, while *C. jejuni* 286 showed low invasion and binding capacity in all tested conditions. Linking phenotypes to proteomics data, several virulence proteins were affected in cell cultured in the presence of l-fucose or d-arabinose (Table 2). In isolate NCTC11168, the protein Cj0608, which is annotated as putative MacC, was 136.1 and 137.7 fold-change upregulated in the presence of l-fucose and d-arabinose, respectively, while no change was observed in isolate *C. jejuni* 286. Authors of previous studies made a clear link between Cj0608, the putative type 1 secretion system MacABC (Cj0606-Cj0608) and the DccSR regulon, which is involved in adaptation to new environments and host colonization of chicken intestine [47,59]. The MacABC system is understudied in *Campylobacter*, however, it is a system that is well conserved and is linked to stress defence and virulence in Gram-negative pathogenic bacteria [[60], [61], [62]]. The putative MacABC system in *Campylobacter* is highly similar to another tripartite efflux pump, the CmeABC system [60]. Notably, the respective intact MacABC and CmeABC membrane protein complexes were recently extracted from *C. jejuni* cells and characterized [63]. For example, both CmeA and MacA belong to the HlyD superfamily and CmeC and MacC are phylogenetically very closely related [63].

**Table 2:** Presence and up or down regulation of potential virulence proteins. Values indicate a fold-change in comparison with MEMα medium. Present indicates that the protein is detected in all tested conditions, but not significantly changed. ND indicates that the protein is not detected.

		l-fucose	d-arabinose	l-fucose	d-arabinose
**Uniprot**	**Protein**	*C. jejuni* NCTC11168	*C. jejuni* NCTC11168	*C. jejuni* 286	*C. jejuni* 286
**Linked to observed Caco-2 invasion and fibronectin binding phenotypes**					
Q0PAQ9	**Cj0608**	136	138	present	present
Q0PBL7	**Peb3/Cj0289c**	present	present	−1.6	−1.8
Q0PC84	**FlgD/Cj0042**	present	present	−2.0	−1.7
Q0P987	**Cj1169c**	present	present	−3.9	−2.5
Q9PID1	**Cj0371**	present	present	1.9	1.5
**Fibronectin binding proteins**					
Q0P8D9	**CadF/Cj1478c**	present	present	present	present
Q0P8X7	**FlpA/Cj1279c**	present	present	present	present
**Presence and absence of virulence proteins**					
Q0P9B5	**WlaN/Cj1139c**	present	present	ND	ND
Q0PAY8	**FlgB/Cj0528c**	present	present	ND	ND
Q0P8S5	**PseD/Cj1333**	present	present	ND	ND
Q0P9B7	**Cj1137c**	present	present	ND	ND
Q0PAJ4	**CipA/Cj0685c**	ND	ND	present	present
**Uniquely up or downregulated virulence proteins**					
Q59296	**KatA/Cj1385**	21.1	present	present	present
Q0P9D0	**PglC/Cj1124c**	10.2	present	ND	ND
Q0P8J7	**Cj1417c**	−67.3	present	present	present
Q0PC12	**Cj0125c**	present	11.1	present	present
Q0PC57	**CdtB/Cj0078c**	present	1.7	present	present
Q0PBF1	**Cj0358**	present	−1.6	present	present
Q0PC58	**CdtC/Cj0077c**	present	present	28.4	present
Q0P8Q4	**CeuE/Cj1355**	present	present	1.5	present
Q0PBE3	**CmeA/Cj0367c**	present	present	present	1.6
Q0P9X8	**Peb1A/Cj0921c**	present	present	present	−2.1

In isolate *C. jejuni* 286, three proteins were uniquely significantly downregulated and one significantly upregulated, Peb3 (adhesin), FlgD (basal-body rod modification protein) and Cj1169c (putative periplasmic protein), and Cj0371 (involved in chemotaxis), respectively (Table 2). Interestingly, Cj0371 is considered an “anti-virulence gene”, as it plays a negative role on *Campylobacters* chemotaxis. In the study of Du et al. (2018), the authors hypothesized that functions encoded by such genes may support colonization and coexistence of *Campylobacter* in animal hosts, including chickens [56]. However, these findings do not directly provide a link with *in vitro* virulence data obtained with *C. jejuni* 286, as cells precultured in MEMα, MEMαF and MEMαA medium showed comparable low Caco-2 invasion and fibronectin binding capacity. Moreover, *C. jejuni* NCTC11168 did not produce CipA, while it produced five virulence proteins that *C. jejuni* 286 did not produce, namely, FlgB, PseD, WlaN, PglC and Cj1137c, with the latter three having roles in lipooligosaccharide (LOS) synthesis or glycosylation. LOS represent an integral component of the *Campylobacter* cell membrane with a structure of core oligosaccharides forming inner and outer core regions and a lipid A moiety, and has been shown to play roles in *Campylobacter* physiology and virulence [[64], [65], [66]].

In conclusion, our study demonstrated that possessing the l-fucose utilization cluster allows *Campylobacter* isolates *C. jejuni* NCTC11168 and *C. jejuni* 286 to metabolize l-fucose and d-arabinose, resulting in enhanced survival for both isolates. An increasing trend for invasion of Caco-2 cells and significantly increased binding to fibronectin after pre-activation with l-fucose or d-arabinose was only found in isolate *C. jejuni* NCTC11168 in the tested conditions. We were able to link the more than 135-fold higher expression of protein Cj0608, the putative TolC-like component MacC, to this observed phenotype. Cj0608 forms together with the detected Cj0606 (MacA) and Cj0607 (MacB) proteins, the tripartite secretion system MacABC, previously shown to operate in diverse cellular processes, including antibiotic resistance, cell envelope stress response and virulence in a range of Gram-negative pathogenic bacteria [[60], [61], [62],^67^]. l-fucose and d-arabinose utilization induced-upregulation of this system, in combination with five virulence proteins uniquely produced by *C. jejuni* NCTC11168, including three enzymes involved in LOS synthesis, WlaN (Beta-1,3 galactosyltransferase), PglC (glycosyltransferase) and Cj1137c (putative glycosyltransferase) [64,66], conceivably affected cell surface structure. Further research is necessary to verify the role of putative MacABC secretion system in modulation of cell surface structures and virulence in *C. jejuni*.

## CRediT authorship contribution statement

**Pjotr S. Middendorf:** Writing – review & editing, Writing – original draft. **Lucas M. Wijnands:** Methodology. **Sjef Boeren:** Methodology. **Aldert L. Zomer:** Writing – review & editing, Supervision, Software. **Wilma F. Jacobs-Reitsma:** Writing – review & editing, Supervision. **Heidy M.W. den Besten:** Writing – review & editing, Supervision, Project administration, Conceptualization. **Tjakko Abee:** Writing – review & editing, Supervision, Methodology, Conceptualization.

## Declaration of competing interest

The authors declare that they have no known competing financial interests or personal relationships that could have appeared to influence the work reported in this paper.
