# Supplementary material for: Activation of the l-fucose utilization cluster in Campylobacter jejuni induces proteomic changes and enhances Caco-2 cell invasion and fibronectin binding
Source: Heliyon. 2024 Jul 26;10(16):e34996. doi: 10.1016/j.heliyon.2024.e34996 (PMC11365321; doi:10.1016/j.heliyon.2024.e34996)
Supplement: Multimedia component 1 [file mmc1.docx]

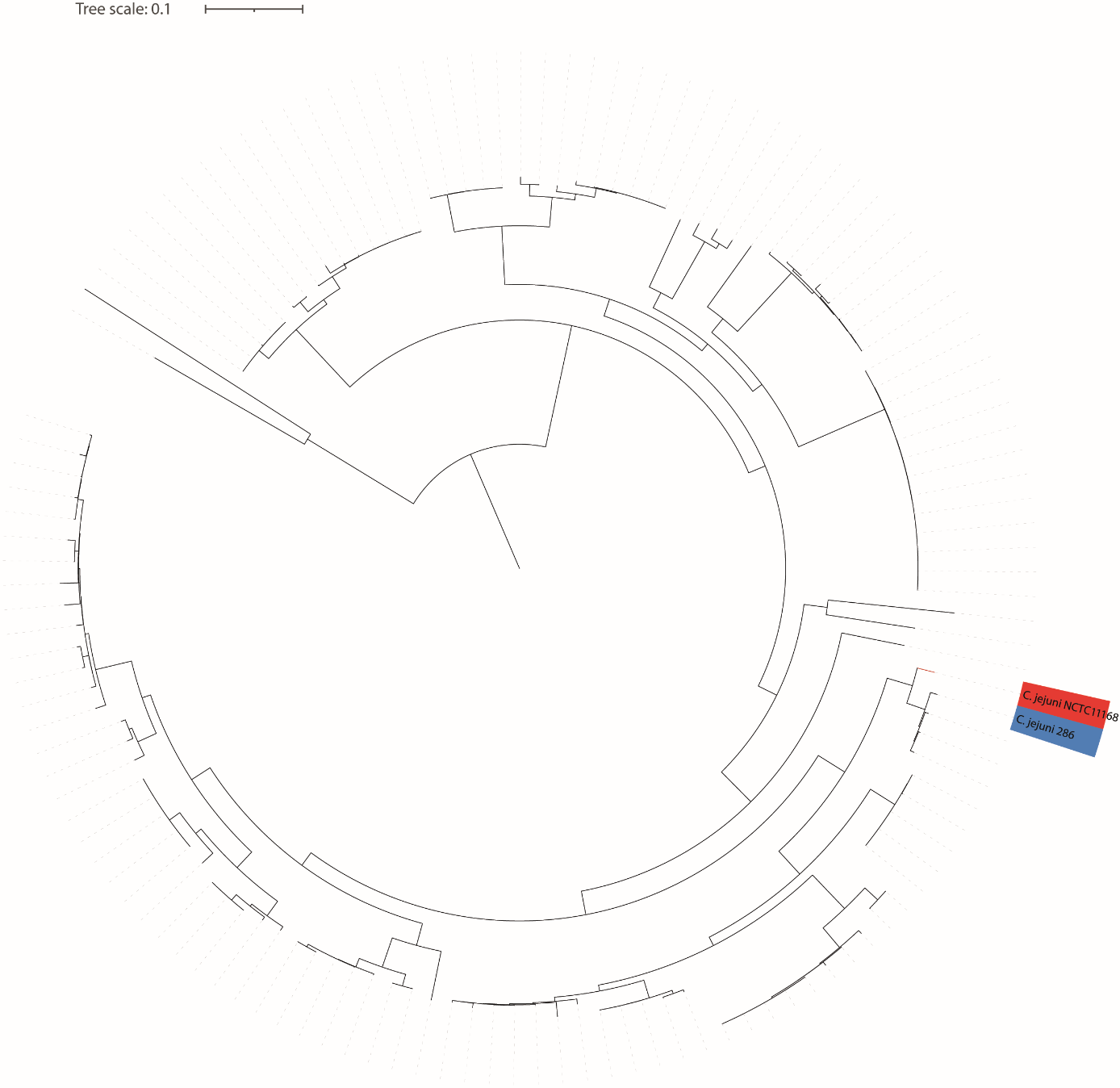


Suppl. Fig. 1. Phylogenetic tree of genomes of human C. jejuni isolates. C. jejuni NCTC11168 and C. jejuni 286 are highlighted in red and blue, respectively.


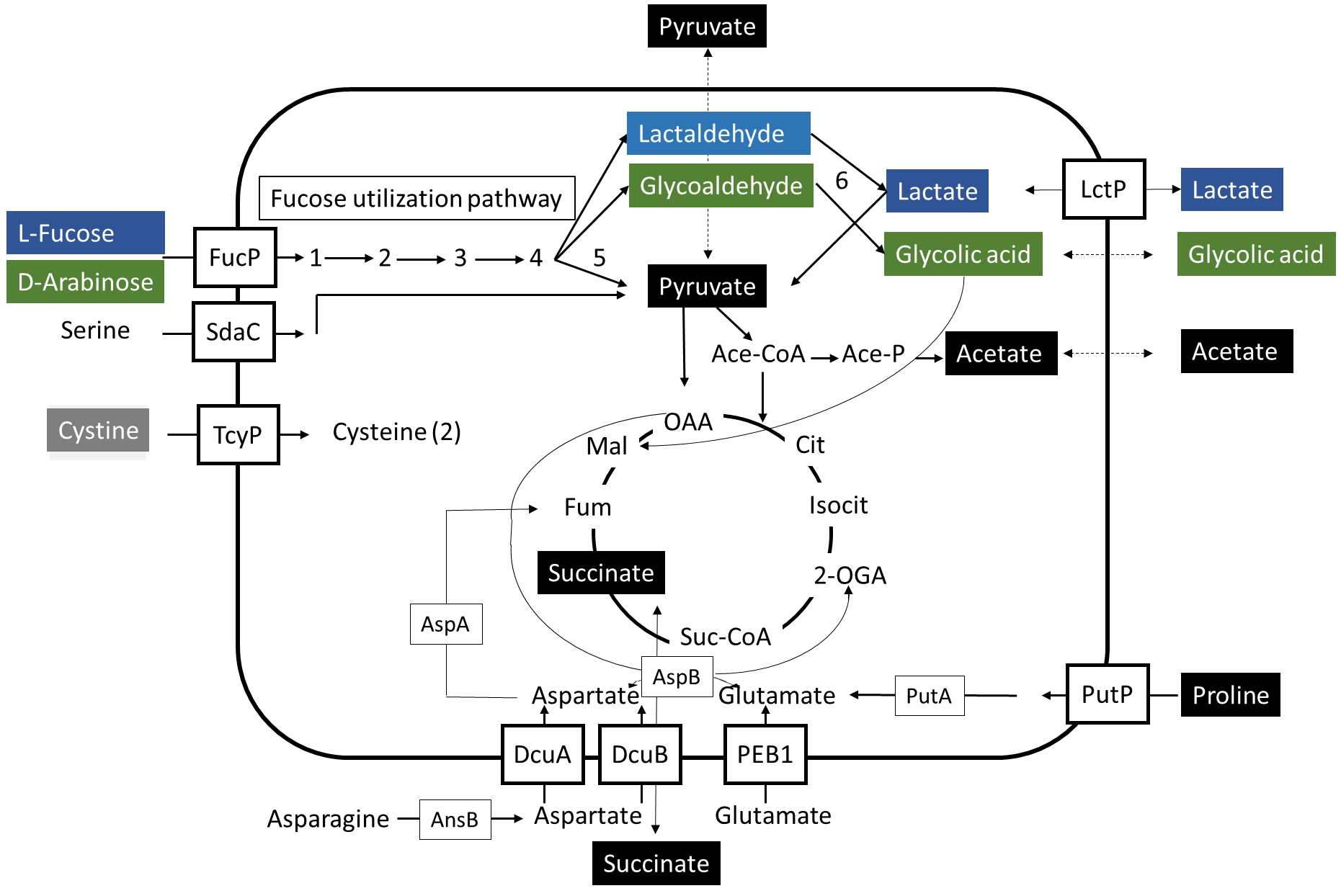


Suppl. Fig. 2. Schematic overview of uptake and metabolism uniquely coupled to fucose (blue) or arabinose (green) utilization in Campylobacter. Bidirectional arrows indicate efflux and uptake/metabolism of lactate in MEMαF, glycolic acid in MEMαA and acetate, pyruvate and succinate in MEMαF and MEMαA. The blue boxes indicate L-fucose transport and metabolism, the green boxes represent D-arabinose transport and metabolism, the black boxes show compounds of which the production and metabolism is linked to L-fucose and D-arabinose metabolism, and the dark grey box displays uptake and intracellular reduction resulting in two (2) cysteines, uniquely linked to L-fucose utilization. Uptake and metabolism of serine, asparagine, aspartate, glutamate, proline and cystine with respective putative transporters are indicated. Figure adapted from [36].

Suppl. Fig. 3. Glycolic acid production in the presence of D-arabinose. Peak area increased over time, indicating a larger amount of glycolic acid in the medium.


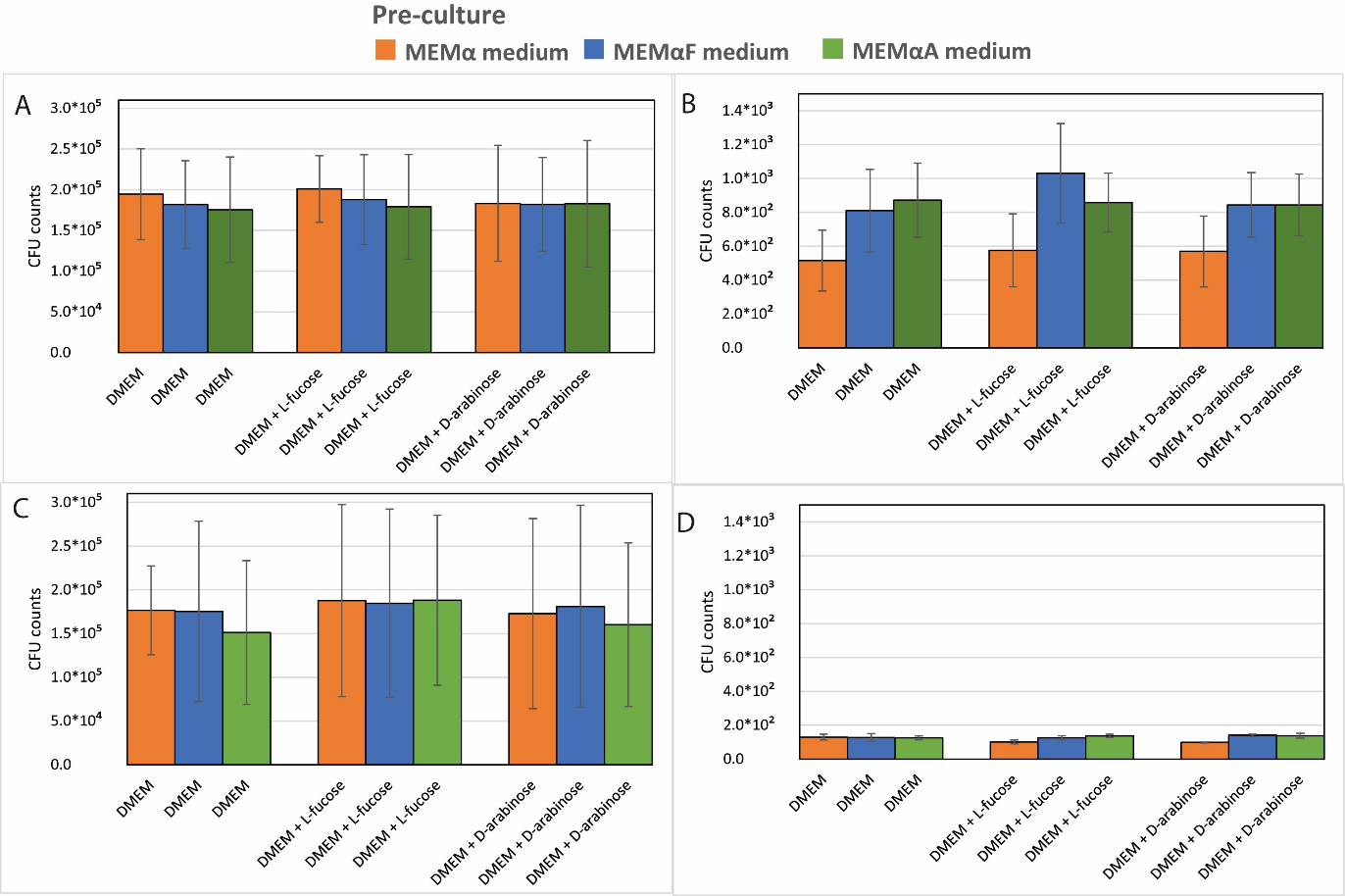


Suppl. Fig. 4. Caco-2 cell adhesion and invasion results of C. jejuni NCTC11168 and C. jejuni 286 that were precultured for two days in MEMα medium (orange bars), MEMαF medium (blue bars) or MEMαA medium (green bars) and incubated in in DMEM, DMEM+ L-fucose or DMEM + D-arabinose. Adhesion (A) and invasion (B) of C. jejuni NCTC11168, and adhesion (C) and invasion (D) of C. jejuni 286. Error bars indicate the standard deviation of three biological replicates.

Suppl. Table 1 Full list of significant up and down regulated proteins (>1.5-fold-change).

| **Protein** | **Function** | **NCTC11168 fuc vs ref** | **NCTC11168 ara vs ref** | **286 fuc vs ref** | **286 ara vs ref** |
| --- | --- | --- | --- | --- | --- |
| Cj0481\|Q0PB32 | Dihydrodipicolinate synthase | 23.6 | 12.4 | 19.5 | 11.8 |
| Cj0482\|Q0PB31 | Altronate hydrolase/dehydratase | 273 | 160 | 152 | 101 |
| Cj0483\|Q0PB30 | Altronate hydrolase/dehydratase | 512 | 253 | 36.2 | 34.2 |
| Cj0484\|Q0PB29 | MFS transporter |  |  | 102 | 69.2 |
| Cj0485\|Q0PB28 | Short chain dehydrogenase | 23.6 | 23.6 | 17.4 | 13.3 |
| Cj0486\|Q0PB27 | MFS transporter | 46.4 | 21.8 | 19.9 | 15.2 |
| Cj0487\|Q0PB26 | Amidohydrolase | 88.0 | 39.6 | 9.0 | 8.6 |
| Cj0488\|Q0PB25 | Epimerase | 70.3 | 39.1 | 140 | 93.3 |
| Cj0489\|Q0PB23 | Aldehyde dehydrogenase | 3.0 |  | 26.6 | 18.1 |
| KatA\|Q59296 | H2O2 detoxification | 21.1 |  |  |  |
| PglC\|Q0P9D0 | Galactosyltransferase | 10.2 |  |  |  |
| Cj1658\|Q0P7X1 | ferri-rhodotorulic acid | 7.6 |  |  |  |
| RpmB\|Q9PI58 | 50S ribosomal protein L28 | 4.1 |  |  |  |
| RpsT\|Q9PM64 | 30S ribosomal protein S20 | 3.7 |  |  |  |
| Cj0771c\|Q0PAB9 | Membrane | 3.5 | 54.4 | 5.6 | 5.8 |
| RpmF\|Q9PIG9 | 50S ribosomal protein L32 | 3.4 |  |  |  |
| RpsS\|Q9PLX5 | 30S ribosomal protein S19 | 2.4 |  |  |  |
| RpmA\|Q9PJ31 | 50S ribosomal protein L27 | 2.3 |  | -2.3 |  |
| RpsI\|Q9PMI3 | 30S ribosomal protein S9 | 2.1 |  |  |  |
| Rp10\|Q0P7U2 | 50S ribosomal protein L15 | 2.0 |  |  |  |
| DsbA\|Q0PA24 | Thiol:disulfide interchange protein DsbA | 1.9 | 1.6 |  |  |
| RplV\|Q9PLX6 | 50S ribosomal protein L22 | 1.9 |  |  |  |
| RpmG\|Q9PI38 | 50S ribosomal protein L33 | 1.8 |  |  |  |
| RplP\|Q9PLX8 | 50S ribosomal protein L16 | 1.8 |  |  |  |
| RpmC\|Q9PLX9 | 50S ribosomal protein L29 | 1.7 |  |  |  |
| Cj0185c\|Q0PBV4 | Putative phnA domain protein | 1.7 | 1.7 |  |  |
| RpsU\|Q9PID2 | 30S ribosomal protein S2 | 1.6 |  |  |  |
| TrpF\|Q9PIF3 | N-(5'-phosphoribosyl)anthranilate isomerase | 1.6 |  |  |  |
| RplU\|Q0PC43 | 50S ribosomal protein L21 | 1.6 | 1.7 |  |  |
| Cj0414\|Q0PB96 | Xidoreductase subunit | 1.6 |  | 2.5 | 2.2 |
| Cj0539\|Q0PAX7 | Uncharacterized protein | -1.5 |  |  |  |
| Cj0413\|Q0PB97 | Beta-lactamase | -1.6 |  | -2.4 |  |
| KdsB\|Q0PA77 | 3-deoxy-manno-octulosonate cytidylyltransferase | -1.6 |  |  |  |
| PutA\|Q0P8B5 | Oxidizes proline to glutamate | -1.7 | -1.6 | -1.6 | -2.2 |
| PurQ\|Q9PHZ7 | Phosphoribosylformylglycinamidine synthase subunit | -1.7 |  |  |  |
| MoaE\|Q0P8A1 | MPT synthase subunit 2 | -2.0 | -1.7 |  |  |
| Cj0375\|Q0PBD5 | putative lipoprotein | -2.1 |  |  |  |
| CstA\|Q0P9Y2 | carbon starvation protein A | -3.2 |  | -1.9 | -2.5 |
| TpiA\|Q9PMQ6 | Putative triosephosphate isomerase | -35.8 | -14.3 |  |  |
| Cj0200c\|Q0PBT9 | putative periplasmic protein | -262 | -99.0 | -115 | -115 |
| Cj0519\|Q0PAZ6 | Putative rhodanese-like domain protein |  | 15.5 |  |  |
| CeuE\|Q0P8Q4 | Enterochelin uptake periplasmic binding protein |  |  | 1.5 |  |
| Cbf2\|Q0PAS1 | peptidyl-prolyl cis-trans isomerase |  |  | 2.0 |  |
| Cj1417c\|Q0P8J7 | Gamma-glutamyl-CDP-amidate hydrolase | -67.3 |  |  |  |
| Cj0415\|Q0PB95 | GMC oxidoreductase subunit |  |  | 2.5 | 2.2 |
| Cj0915\|Q0P9Y4 | Putative hydrolase |  |  | 1.6 | 1.8 |
| AcsA\|Q9PMD2 | Acetyl-coenzyme A synthetase |  |  | -1.6 | -2.7 |
| FabD\|Q0PC21 | Malonyl CoA-acyl carrier protein transacylase |  |  |  | -1.6 |
| MetAA\|Q9PLV2 | Homoserine O-acetyltransferase |  |  | 1.7 | 1.5 |
| MetE\|Q9PN94 | homocysteine methyltransferase |  |  | 1.7 | 1.8 |
| Cj0653c\|Q0PAL5 | Putative aminopeptidase |  |  | -3.2 | -4.5 |
| CfbpC\|Q0PBW6 | Putative iron-uptake ABC transport system |  |  | -1.6 |  |
| HydA\|Q0P8Y9 | Ni/Fe-hydrogenase small chain |  | 1.6 |  |  |
| HydC\|Q0P8Z1 | Ni/Fe-hydrogenase B-type cytochrome subunit |  | -40.0 |  |  |
| SdhC\|Q0PB71 | Putative succinate dehydrogenase subunit C |  | -1.8 |  |  |
| LeuB\|Q9PLW0 | 3-isopropylmalate dehydrogenase |  |  |  | 1.6 |
| Cj0264c\|Q0PBP1 | Trimethylamine-N-oxide reductase |  |  |  | 1.5 |
| Cj0075c\|Q0PC60 | Putative oxidoreductase iron-sulfur subunit |  |  |  | -1.6 |
| LctP\|Q0PC21 | Malonyl CoA-acyl carrier protein transacylase |  |  |  | -1.6 |
| Cj1515c\|Q0P8A4 | Carboxynorspermidine/carboxyspermidine decarboxylase |  |  |  | -1.6 |
| Cj0073c\|Q0PC62 | LUD_dom domain-containing protein |  |  |  | -1.5 |
| Cj1653c\|Q0P7X5 | Putative lipoprotein |  | 1.8 |  |  |
| Cj0599\|Q0PAR8 | Putative OmpA family membrane protein |  | -1.5 |  |  |
| Peb3\|Q0PBL7 | Major antigenic peptide |  |  | -1.6 | -1.8 |
| Cj0093\|Q0PC44 | Putative periplasmic protein |  |  |  | 1.5 |
| Cj0455c\|Q0PB58 | Putative membrane protein |  |  | -40.1 |  |
| FlgD\|Q0PC84 | Basal-body rod modification protein FlgD |  |  | -2.0 | -1.7 |
| PrmA\|Q9PNH7 | Ribosomal protein L11 methyltransferase |  |  | -30.4 |  |
| Cj0722c\|Q0PAF7 | Putative DNA methylase |  |  |  | -78.5 |
| Cj0358\|Q0PBF1 | Putative cytochrome C551 peroxidase |  | -1.6 |  |  |
| Cj1289\|Q0P8W8 | Possible periplasmic protein |  |  |  | 1.6 |
| Cj0761\|Q0PAC9 | possibly membrane related |  | 54.4 |  |  |
| CdtB\|Q0PC57 | Cytolethal distending toxin B |  | 1.7 |  |  |
| RplQ\|Q0P832 | 50S ribosomal protein L17 |  |  | 1.8 |  |
| Cj0144\|Q0PBZ3 | Methyl-accepting chemotaxis signal transduction protein |  |  | -10.6 | -10.6 |
| Cj0834c\|Q0PA56 | Ankyrin repeat-containing putative periplasmic protein |  |  |  | -1.5 |
| Peb1A\|Q0P9X8 | Major cell-binding factor ABC-transporter |  |  |  | -2.1 |
| CmeA\|Q0PBE3 | Periplasmic fusion protein CmeA |  |  |  | 1.6 |
| CJ1232\|Q0P924 | Uncharacterized protein |  | 15.0 |  |  |
| Cdtc\|Q0PC58 | Cytolethal distending toxin C |  |  | 28.4 |  |
| Cj0092\|Q0PC45 | Putative periplasmic protein |  |  | 3.8 | 2.5 |
| Cj1666c\|Q0P7W3 | periplasmic protein |  |  | 2.3 |  |
| Cj0371\|Q9PID1 | lipoprotein |  |  | 1.9 | 1.5 |
| Cj0998c\|Q0P9Q4 | utative periplasmic protein |  |  | 1.5 |  |
| Cj1169c\|Q0P987 | Putative periplasmic protein |  |  | -3.9 | -2.5 |
| Cj1650\|Q0P7X8 | Uncharacterized protein |  |  | -1.6 |  |
| Cj1623\|Q0P805 | Putative membrane protein |  |  |  | -8.6 |
|  |  |  |  |  |  |

Suppl. Table 2 Full presence/absence list of 236 potential virulence proteins in C. jejuni NCTC11168 and 286. Presence and up or down regulation of potential virulence proteins in MEMαF and MEMαA grown cells is indicated, with fold-change in comparison to cells in MEMα. Present, indicates that the protein is detected in tested conditions, but not significantly changed; ND indicates that the protein is not detected in tested conditions.

|  |  | **L-fucose** | **D-arabinose** | **L-fucose** | **D-arabinose** |
| --- | --- | --- | --- | --- | --- |
| **Uniprot** | **Protein** | ***C. jejuni* NCTC11168** | ***C. jejuni* NCTC111682** | ***C. jejuni* 286** | ***C. jejuni* 286** |
| Q0P8D9 | **CadF/Cj1478c** | present | present | present | present |
| Q0P8X7 | **FlpA/Cj1279c** | -1.3 | present | present | present |
| Q0PAY8 | **FlgB/Cj0528c** | present | present | ND | ND |
| Q9PHP0 | **Cj0627/capA** | present | present | ND | ND |
| Q0P8S5 | **PseD/Cj1333** | present | present | ND | ND |
| Q0P9B5 | **WlaN/Cj1139c** | present | present | ND | ND |
| Q0P9B7 | **Cj1137c** | present | present | ND | ND |
| Q0P9D0 | **PglC/Cj1124c** | 10.2 | present | ND | ND |
| Q0PAJ4 | **CipA/Cj0685c** | ND | ND | present | present |
| Q0P9Y2 | **CstA/Cj0917c** | -3.2 | present | -1.9 | -2.5 |
| Q0PC12 | **Cj0125c** | present | 11.1 | present | present |
| Q0P8J9 | **Cj1415c** | -1.2 | present | present | present |
| Q0P8W3 | **PseC/Cj1294** | 1.2 | present | present | present |
| Q0P863 | **PlfA/Cj1565c** | -1.1 | present | present | present |
| Q0P8Z5 | **RacR/Cj1261** | 1.2 | present | present | 1.4 |
| Q0P8X7 | **CJE1415/Cj1279c** | -1.3 | present | present | present |
| Q59296 | **KatA/Cj1385** | 21.1 | present | present | present |
| Q0P8J7 | **Cj1417c** | -67.3 | present | present | present |
| Q9PN97 | **LuxS/Cj1198** | present | -1.3 | present | present |
| Q0P8I5 | **Cj1429c** | present | 1.2 | present | present |
| Q0P9C9 | **PglA/Cj1125c** | present | -1.3 | present | present |
| Q0P8U1 | **PseA/Cj1316c** | present | present | 1.1 | present |
| Q0P8H1 | **Cj1443c** | present | -1.2 | present | present |
| Q0P8R0 | **CjE1538** | present | -1.3 | present | present |
| Q0PBF1 | **Cj0358** | present | -1.6 | present | present |
| Q0PC57 | **CdtB/Cj0078c** | present | 1.7 | present | present |
| Q0PC58 | **CdtC/Cj0077c** | present | present | 28.4 | present |
| Q0P9X8 | **Peb1A/Cj0921c** | present | present | present | -2.1 |
| Q0PBL7 | **Peb3/Cj0289c** | present | present | -1.6 | -1.8 |
| P96747 | **FlaC/Cj0720c** | present | present | present | 1.4 |
| Q0P7V5 | **MqnD/Cj1674** | present | present | present | 1.4 |
| Q0P8T3 | **Cj1324** | present | present | -1.5 | present |
| Q0PBE3 | **cmeA/Cj0367c** | present | present | 1.4 | 1.6 |
| Q9PID1 | **Cj0371** | present | present | 1.9 | 1.5 |
| Q0P8Q4 | **CeuE/Cj1355** | present | present | 1.5 | present |
| Q0P8H7 | **Cj1437c** | present | present | -1.3 | present |
| Q0PC84 | **FlgD/Cj0042** | present | present | -2.0 | -1.7 |
| Q0P7Q2 | **FlgE/Cj0043** | present | present | -1.3 | -1.3 |
| Q0PBU4 | **FliI/Cj0195** | present | present | present | 1.3 |
| P96747 | **FlaC/Cj0720c** | present | present | present | 1.4 |
| Q0P7Q2 | **FlgE2/Cj1729c** | present | present | -1.3 | -1.3 |
| Q0P987 | **Cj1169c** | present | present | -3.9 | -2.5 |
| Q0PBH4 | **FlhB/Cj0335** | present | present | present | present |
| Q0PAY9 | **FlgC/Cj0527c** | present | present | present | present |
| Q9PHW6 | **FliD/Cj0548** | present | present | present | present |
| Q0PAI1 | **FlgG/Cj0698** | present | present | present | present |
| Q0P7Y2 | **IamB/Cj1646** | present | present | present | present |
| Q0PC56 | **CdtA/Cj0079c** | present | present | present | present |
| O85213 | **DnaJ/Cj1260c** | present | present | present | present |
| Q0P9Y5 | **CiaB/Cj0914c** | present | present | present | present |
| Q0P8D9 | **CadF/T00461** | present | present | present | present |
| Q0PB65 | **AcfB/Cj0448c** | present | present | present | present |
| Q0P966 | **CetA/Cj1190c** | present | present | present | present |
| Q0P9X5 | **CheB/Cj0924c** | present | present | present | present |
| Q0P9X6 | **CheR/Cj0923c** | present | present | present | present |
| Q0PBM1 | **CheV/Cj0285c** | present | present | present | present |
| Q0PBM3 | **cheW/Cj0283c** | present | present | present | present |
| P0C635 | **CheY/Cj1118c** | present | present | present | present |
| Q0P8B2 | **Tlp1/Cj1506c** | present | present | present | present |
| Q0PCA6 | **Tlp10/Cj0019c** | present | present | present | present |
| Q0PA15 | **FlhA/Cj0882c** | present | present | present | present |
| Q0PA70 | **FliP/Cj0820c** | present | present | present | present |
| Q0P928 | **HtrA/Cj1228c** | present | present | present | present |
| Q0P8T2 | **Cj1325** | present | present | present | present |
| Q9PMJ8 | **FlgI/Cj1462** | present | present | present | present |
| Q0PC72 | **FliA/Cj0061c** | present | present | present | present |
| Q0PBJ1 | **FliF/Cj0318** | present | present | present | present |
| Q0PC73 | **FliM/Cj0060c** | present | present | present | present |
| Q0PC74 | **FliY/Cj0059c** | present | present | present | present |
| Q0PAK4 | **RpoN/Cj0670** | present | present | present | present |
| Q0PBE4 | **CmeB/Cj0366c** | present | present | present | present |
| Q0PBE5 | **CmeC/Cj0365c** | present | present | present | present |
| Q0PBE2 | **CmeR/Cj0368c** | present | present | present | present |
| Q0PBH5 | **AhpC/Cj0334** | present | present | present | present |
| Q0PCB3 | **Rrc/Cj0012c** | present | present | present | present |
| Q0PCA5 | **Cj0020c** | present | present | present | present |
| Q0P8Y4 | **SpoT/Cj1272c** | present | present | present | present |
| Q9PPE0 | **Tpx/Cj0779** | present | present | present | present |
| Q0PB65 | **Cj0448c** | present | present | present | present |
| P45492 | **JlpA** | present | present | present | present |
| P80672 | **PorA/Cjj81176** | present | present | present | present |
| Q0P8K1 | **Cj1413c** | present | present | present | present |
| Q0P8K0 | **Cj1414c** | present | present | present | present |
| Q0P8J8 | **Cj1416c** | present | present | present | present |
| Q0P8J6 | **Cj1418c** | present | present | present | present |
| Q0P8J5 | **Cj1419c** | present | present | present | present |
| Q0P8J4 | **Cj1420c** | present | present | present | present |
| Q0P8J3 | **Cj1421c** | present | present | present | present |
| Q0P8J2 | **Cj1422c** | present | present | present | present |
| Q0P8J1 | **Cj1423c** | present | present | present | present |
| Q9PMN3 | **Cj1424c** | present | present | present | present |
| Q0P8I9 | **Cj1425c** | present | present | present | present |
| Q0P8I8 | **Cj1426c** | present | present | present | present |
| Q0P8I7 | **Cj1427c** | present | present | present | present |
| Q0P8I6 | **Cj1428c** | present | present | present | present |
| Q0P8I4 | **Cj1430c** | present | present | present | present |
| Q0P8I3 | **Cj1431c** | present | present | present | present |
| Q0P8I2 | **Cj1432c** | present | present | present | present |
| Q0P8I1 | **Cj1433c** | present | present | present | present |
| Q0P8I0 | **Cj1434c** | present | present | present | present |
| Q0P8H9 | **Cj1435c** | present | present | present | present |
| Q0P8H8 | **Cj1436c** | present | present | present | present |
| Q0P8H6 | **Cj1438c** | present | present | present | present |
| Q0P8H5 | **Cj1439c** | present | present | present | present |
| Q0P8H4 | **Cj1440c** | present | present | present | present |
| Q0P8H3 | **Cj1441c** | present | present | present | present |
| Q0P8H2 | **Cj1442c** | present | present | present | present |
| Q0P8H1 | **Cj1444c** | present | present | present | present |
| Q0P8H0 | **Cj1445c** | present | present | present | present |
| Q0P8G8 | **Cj1447c** | present | present | present | present |
| Q0P9D5 | **PglG/Cj1119c** | present | present | present | present |
| Q0P9D4 | **PglF/Cj1120c** | present | present | present | present |
| Q0P9D3 | **PglE/Cj1121c** | present | present | present | present |
| Q0P9D1 | **PglD/Cj1123c** | present | present | present | present |
| Q0P9C8 | **PglB/Cj1126c** | present | present | present | present |
| Q0P9C7 | **PglJ/Cj1127c** | present | present | present | present |
| Q0P9C6 | **PglI/Cj1128c** | present | present | present | present |
| Q0P9C5 | **PglH/Cj1129c** | present | present | present | present |
| Q0P8W4 | **PseB/Cj1293** | present | present | present | present |
| Q0P8U6 | **PseF/Cj1311** | present | present | present | present |
| Q0P8U5 | **PseG/Cj1312** | present | present | present | present |
| Q0P8U4 | **PseH/Cj1313** | present | present | present | present |
| Q0P8U0 | **PseI/Cj1317** | present | present | present | present |
| Q0P8T1 | **NeuB2/Cj1327** | present | present | present | present |
| Q0P8T0 | **NeuC2/Cj1328** | present | present | present | present |
| Q0P8S7 | **PtmB/Cj1331** | present | present | present | present |
| Q0P8S6 | **PtmA/Cj1332** | present | present | present | present |
| Q0P8S2 | **PseE/Cj1337** | present | present | present | present |
| Q0P8S3 | **Maf1/Cj1318** | present | present | present | present |
| Q0P8S3 | **Maf4/Cj1336** | present | present | present | present |
| Q0P8R9 | **Maf6/Cj1340c** | present | present | present | present |
| Q0P8R8 | **Maf7/Cj1341c** | present | present | present | present |
| Q0PC70 | **FlhG/Cj0063c** | present | present | present | present |
| O52908 | **FlhF/Cj0064c** | present | present | present | present |
| Q0PBJ0 | **FliG/Cj0319** | present | present | present | present |
| Q0PBI9 | **FliH/Cj0320** | present | present | present | present |
| Q0PBH3 | **MotB/Cj0336c** | present | present | present | present |
| Q0PBH2 | **MotA/Cj0337c** | present | present | present | present |
| Q0PBF8 | **FliN/Cj0351** | present | present | present | present |
| Q9PHY8 | **FliE/Cj0526c** | present | present | present | present |
| Q0PAW9 | **FlaG/Cj0547** | present | present | present | present |
| Q0P9M8 | **FlgR/Cj1024c** | present | present | present | present |
| P56964 | **FlaB/Cj1338c** | present | present | present | present |
| P56963 | **FlaA/Cj1339c** | present | present | present | present |
| Q0P8K6 | **FliL/Cj1408** | present | present | present | present |
| Q0P8E9 | **FlgK/Cj1466** | present | present | present | present |
| Q0P8Z4 | **RacS/Cj1262** | present | present | present | present |
| Q0PC46 | **Cj0091** | present | present | present | present |
| Q0PAS9 | **TylA/cj0588** | present | present | present | present |
| Q0P9Y5 | **CiaB/Cj0914c** | present | present | present | present |
| Q0P967 | **CetB/Cj1189c** | present | present | present | present |
| Q0PBM2 | **CheA/Cj0284c** | present | present | present | present |
| Q0P8S4 | **Maf3/Cj1334** | present | present | present | present |
| Q0P9C1 | **C8J/1073/WaaC** | present | present | present | present |
| Q0P9A9 | **C8J/1091/WaaV** | present | present | present | present |
| Q0P9A8 | **C8J/1092/WaaF** | present | present | present | present |
| Q9PNE6 | **C8J/1095/GmhA1** | present | present | present | present |
| Q6TG09 | **C8J/1096/WaaE** | present | present | present | present |
| Q0PA08 | **CJE0969/Cj0890c** | present | present | present | present |
| Q0P934 | **CJE1357/DccS** | present | present | present | present |
| Q0P933 | **CJE1358/DccR** | present | present | present | present |
| Q0P930 | **CJE1361/Cj1226c** | present | present | present | present |
| Q0P929 | **CJE1362/Cj1227c** | present | present | present | present |
| Q0P8C5 | **CjE1665/Cj1492c** | present | present | present | present |
| Q0P914 | **Cj1242/CiaC** | present | present | present | present |
| Q0P9I5 | **Cj1069** | present | present | present | present |
| Q0P870 | **Cj1556** | present | present | present | present |
| Q0P8K4 | **Cj1410c** | present | present | present | present |
| Q0P9S5 | **Cj0977** | present | present | present | present |
| Q9PM85 | **InfA/Cj1590** | present | present | present | present |
| Q0P8N8 | **Cj1371** | present | present | present | present |
| Q0P9L0 | **Cj1042c** | present | present | present | present |
| Q0PC83 | **FlgE** | ND | ND | ND | ND |
| Q0PAW7 | **FliS/Cj0549** | ND | ND | ND | ND |
| Q0PA11 | **FlaD/Cj0887c** | ND | ND | ND | ND |
| Q0P8Q8 | **PldA** | ND | ND | ND | ND |
| Q0P7V4 | **FliQ/Cj1675** | ND | ND | ND | ND |
| Q0P977 | **FliR/Cj1179c** | ND | ND | ND | ND |
| Q0PAD4 | **CfrA/Cj0755** | ND | ND | ND | ND |
| Q0P814 | **ChuA/Cj1614** | ND | ND | ND | ND |
| Q0PBW1 | **Cj0178** | ND | ND | ND | ND |
| Q0P8T6 | **Cj1321** | ND | ND | ND | ND |
| Q0P8T4 | **Cj1323** | ND | ND | ND | ND |
| Q9PPM0 | **FgH/Cj0687c** | ND | ND | ND | ND |
| Q0PC85 | **FliK/Cj0041** | ND | ND | ND | ND |
| Q0P8G8 | **TagH/CCC13826** | ND | ND | ND | ND |
| Q0P8T6 | **Cj1321** | ND | ND | ND | ND |
| Q0P8G7 | **Cj1448c** | ND | ND | ND | ND |
| Q0P9C0 | **C8J/1074/HtrB** | ND | ND | ND | ND |
| Q0PC85 | **Cj0041** | ND | ND | ND | ND |
| Q0PAI2 | **FlgG2/Cj0697** | ND | ND | ND | ND |
| Q0P8C6 | **CjE1664/Cj1491c** | ND | ND | ND | ND |
| Q0P7V2 | **Cj1677/CapB** | ND | ND | ND | ND |
